# Effects of the COVID-19 pandemic on early infant diagnosis of HIV in Cape Town, South Africa

**DOI:** 10.4102/sajhivmed.v25i1.1542

**Published:** 2024-03-18

**Authors:** Hendrike van Vollenhoven, Emma Kalk, Stuart M. Kroon, Tafadzwa Maseko, Florence Phelanyane, Jonathan Euvrard, Lezanne Fourie, Nicolene le Roux, Phumza Nongena

**Affiliations:** 1 Department of Paediatrics and Child Health, Faculty of Health Sciences, University of Cape Town, Cape Town, South Africa; 2 School of Public Health, Faculty of Health Science, University of Cape Town, Cape Town, South Africa; 3 Family Medicine and Population Health (FAMPOP), Department of Epidemiology, Faculty of Medicine and Health Sciences, University of Antwerp, Antwerp, Belgium; 4 Health Intelligence Directorate, Western Cape Department of Health, Cape Town, South Africa; 5 Department of Neonatology, Mowbray Maternity Hospital, Cape Town, South Africa; 6 HIV/AIDS, STI’s and Tuberculosis Directorate (HAST), New Somerset Hospital, Cape Town, South Africa

**Keywords:** vertical transmission prevention, VTP, HIV, COVID-19, early infant diagnosis, vertical transmission, prevention of mother-to-child transmission, PMTCT

## Abstract

**Background:**

In South Africa, infants who are HIV-exposed are tested for HIV at birth and 10 weeks of age. The COVID-19 pandemic lockdown restrictions resulted in reduced access to healthcare services and uncertain impact on early infant HIV testing.

**Objectives:**

To describe the effects of the COVID-19 pandemic lockdown restrictions on early infant HIV testing and diagnosis in Cape Town, South Africa.

**Method:**

This retrospective cohort study compares HIV-exposed infants born during the first COVID-19 pandemic lockdown (2020) to those born in the same period the year before (2019). Laboratory and other data were abstracted from the Provincial Health Data Centre.

**Results:**

A total of 2888 infants were included: 1474 born in 2020 and 1413 in 2019. Compared to 2019, there was an increase in the 10-week HIV polymerase chain reaction (PCR) uptake in 2020 (71% vs. 60%, *P* < 0.001). There was also an increase in the proportion of infants who demised without 10-week testing or were lost to follow-up in 2020 compared to 2019 (8% vs. 5%, *P* = 0.017). Differences detected in birth HIV PCR positivity rates between the two groups (1.1% vs. 0.5%, *P* = 0.17) did not reach statistical significance; however, a significant increase in vertical transmission of HIV by 10 weeks old was found in the 2020 cohort (1.2% vs. 0.5%. *P* = 0.046).

**Conclusion:**

Vertical transmission of HIV at 10 weeks increased in the Cape Town Metropolitan during the initial COVID-19 lockdown. There was also an increase in the proportion of deaths without testing by 10 weeks in the 2020 group.

**What this study adds:** Preserved rates of infant HIV testing highlighted the resilience of the South African Vertical Transmission Prevention programme. However, increases in vertical transmission and in the proportion of demise and loss to follow-up in 2020 suggests that the pandemic and lockdown restrictions did have a significant impact on access to healthcare.

## Introduction

Untreated HIV infection in infancy results in high mortality and life-long morbidity.^1,2,3,4,5^ Even short delays in treatment correlate with significant disease progression and poorer immune and clinical recovery when treatment is commenced.^4,5,6^ Early and repeated testing of HIV-exposed infants is crucial – diagnosis is key to improving access to timely treatment.^1,7,8,9,10^ The South African Vertical Transmission Prevention (VTP) programme now includes universal HIV polymerase chain reaction (HIV PCR) testing for all HIV-exposed infants at birth and 10 weeks.^11,12^ This emphasis on infant testing is coupled with rapid treatment initiation targets: 7 days from diagnosis to treatment start, with same-day initiation encouraged in infants who are otherwise well.^11,12,13^ The VTP programme has become an essential and effective intervention in reducing childhood HIV infections and HIV-associated mortality.^14,15,16,17,18^

Although universal birth HIV PCR testing has improved infant diagnosis,^8,9,19^ concerns remain about high attrition rates from the VTP programme with many infants being lost to follow-up or not returning for test results.^8,20,21,22,23^ Evidence from countries across Africa, including South Africa, identify numerous barriers to care ranging from healthcare infrastructure and system challenges to socio-economic factors and cultural norms.^20,21,22,24^

On 11 March 2020, the novel coronavirus (SARS-CoV-2) outbreak was declared a global pandemic by the World Health Organization.^25^ By 23 March 2020, South Africa had 402 confirmed cases and the government announced a strict national lockdown which was enforced 4 days later.^26^ The lockdown measures included a complete ban of any activity not deemed essential.^26,27,28,29,30,31^ Despite not being formally suspended, the lockdown led to markedly reduced use of available healthcare services in South Africa.^26^ Economic hardship, restrictions on the freedom of movement and public transport, as well as psychosocial impacts such as fear of infection and strong stay-at-home messaging, are thought to have contributed to decreased uptake of available healthcare services and exacerbation of pre-existing barriers to care.^26,32,33,34,35^

Data show that globally, and in South Africa, immunisation rates dropped, the number of child health visits decreased and there were fewer perinatal care enrolments.^29,30,36,37,38^ Although there was initial evidence of decreased HIV testing nationally and concerns regarding an increase in the absolute number of infants testing HIV-positive at birth,^30,33,34,39^ these findings were not confirmed in subsequent local studies.^37,40,41^

To our knowledge there are no studies that looked at the impact of the COVID-19 pandemic on infant HIV diagnostic services in the Western Cape – one of the regions most severely affected by the early waves of the COVID-19 pandemic.^28^ This study aimed to describe the effects of initial waves of the COVID-19 pandemic and associated lockdown regulations on early infant testing and diagnosis of HIV (EID) in Cape Town, South Africa, compared to the same period the year before.

The objectives were to determine the proportion of HIV-exposed infants born during the COVID-19 lockdown who had birth and 10-week HIV testing compared to infants born in the same period in 2019, before the pandemic, and to determine the proportion of infants testing HIV-positive in each cohort.

## Methods

This was a retrospective cohort study of 2888 infants from two regional hospitals in Cape Town, South Africa.

### Setting and participants

We included routinely collected data from two level 2 hospitals offering obstetric services in the urban Cape Metro health district: Mowbray Maternity Hospital (MMH) and New Somerset Hospital (NSH).

The Cape Metropolitan (Metro) health district serves over 4 million people,^42^ and MMH and NSH manage approximately 11 000 and 6000 deliveries per year, respectively, representing approximately 45% of infants born in the district.^43^ Both hospitals offer primary level care to women in their immediate geographic service area and secondary level care to women living in a wider catchment area if referred by a primary care facility for a pregnancy or delivery deemed high risk. Estimated antenatal HIV prevalence in this population is between 16% and 20%.^44^

Immediate post-natal care of mother-infant dyads (including birth HIV PCR testing and initiation of infant chemoprophylaxis) occurs in hospital. Both study sites offer intensive care unit (ICU)-level care to neonates, and even critically unwell infants would undergo birth PCR testing at the birth facility. Follow-up care after discharge is provided in the community at local primary care facilities. In the case of infants who remain hospitalised, chemoprophylaxis, routine HIV testing and antiretroviral treatment (ART) initiation are provided in hospital.

### Sample size

All HIV-exposed babies born at the selected facilities between 1 March 2020 and 31 August 2020 (during the first COVID-19 lockdown) were compared to those born during the same period in the preceding year, 1 March 2019 to 31 August 2019. This period spans the initial strictest lockdown levels and the peak of the first wave of the COVID-19 pandemic. Power calculations using estimated sample size, known baseline HIV prevalence, vertical transmission risk and baseline uptake of infant HIV PCR testing predicted the study would have 90% power to detect 5% difference in testing uptake and 80% power to detect 1% difference in positive test results.

### Selection criteria

All live-born infants of women living with HIV (WLHIV) at the time of delivery or diagnosed within 7 days of delivery were included. Infants of mothers with negative, discordant, indeterminate or unknown HIV status were excluded. Infants born outside of the study sites (e.g., infants presenting to study sites after delivery) were excluded.

### Data sources and collection

De-identified individualised data were requested from the Provincial Health Data Centre (PHDC). As described by Boulle et al. in their 2019 data centre profile, the PHDC consolidates all person-level data from the Department of Health (DOH) using multiple sources including digital hospital information systems, national laboratory result systems and electronic registers for high priority health conditions such as HIV.^45^ Data are linked through the use of a unique personal identifying folder number which is ubiquitous across the provincial health platforms.^45^

Data were extracted on infant gender, date of birth, gestational age, facility of birth, date and results of first HIV PCR, as well as dates, facilities and results of any subsequent HIV PCR testing up to 14 weeks of age. Vital status was determined using dates of death, for infants known to have demised, and evidence of healthcare access after 10 weeks of age as a proxy in other infants. Infants with no recorded date of death and no evidence of health-care contact after 10 weeks of age were deemed lost to follow-up. Maternal data were extracted on timing of ART initiation in relation to pregnancy.

Aggregate data were also collected from the hard-copy facility-based birth HIV PCR registers at each study site for comparison with the number of birth HIV PCRs captured in the electronic data set.

### Testing definitions

We defined a birth HIV PCR as a test that was taken within 7 days of birth and a 10-week HIV PCR as a test taken between 6 and 14 weeks of birth. Therefore, the age at testing refers to chronological age of the infant.

### Data analysis

The data set was prepared and analysed using R.^46^ Duplicate entries with identical study numbers and birth dates were consolidated. All birth HIV PCR tests were included for analysis. Infants testing HIV-positive at birth were excluded from analysis of 10-week testing. Facility-level data were analysed separately from data obtained from the electronic database to allow for comparative analysis.

Continuous variables were interpreted using means and confidence intervals for normally distributed data or medians and interquartile ranges for non-normally distributed data. Categorical data were described using proportions and frequency tables. The Chi-square test (or, when appropriate, the Fisher’s exact test) was used to test significance and a *P*-value of < 0.05 was considered statistically significant.

### Bias and confounders

To minimise possible selection bias, we included all infants meeting inclusion criteria at both study sites. As this study did not include clinical record review, we could not control for potential confounding variables such as socio-economic circumstances, maternal age, vertical transmission risk stratification, maternal viral load, etc. We aimed to keep groups comparable by keeping the study sites and time periods the same in both years of study. Sample bias may have occurred as all the infants in our cohort were born at regional level referral hospitals in a single health district.

## Results

### Sample characteristics

We enrolled 2888 live-born infants of WLHIV across the two study sites and periods, 1413 born between 1 March 2019 and 31 August 2019 and 1475 in the same period in 2020, during the COVID-19 pandemic lockdown.

Baseline characteristics of the two cohorts were similar (Table 1). The proportion of deliveries at each facility remained consistent with approximately two-thirds of infants being born at MMH and the remaining third at NSH. Gestational ages of infants at birth were similar in both time periods, with most infants being born at term. There were no significant differences in maternal ART coverage between the two time periods.

**TABLE 1:** Baseline characteristics.

Characteristics	Before lockdown (2019) (*n* = 1413)		During lockdown (2020) (*n* = 1475)		Total (*N* = 2888)		*P*
	*n*	%	*n*	%	*n*	%	
**Infant gender†**							
Female	666	47.2	749	50.8	1415	49.0	0.052
Male	746	52.8	726	49.2	1472	51.0	-
**Pregnancy outcome facility**							
Mowbray Maternity Hospital	934	66.1	974	66.1	1908	66.1	0.970
New Somerset Hospital	479	33.9	501	33.9	980	33.9	-
**Gestational age (weeks)**							
< 32	39	2.8	37	2.5	76	2.6	0.895
32–36	243	17.2	250	16.9	493	17.1	-
≥ 37	1131	80	1188	80.5	2319	80.3	-
**Maternal ART status**							
On ART before pregnancy	910	64.4	995	67.5	1905	66	0.341
Re-initiated during pregnancy	425	30.0	399	27.0	824	28.5	-
Newly initiated in pregnancy	63	4.5	65	4.4	128	4.4	-
Initiated after pregnancy	15	1.1	16	1.1	31	1.1	-

ART, antiretroviral treatment.

† , In the 2019 cohort, one infant had missing gender information.

### HIV PCR uptake

There were no significant differences in birth HIV PCR testing uptake which remained over 80% in both years based on the electronic data (Table 2). However, 10-week HIV PCR uptake significantly increased in 2020. In both groups > 90% of infants had at least one HIV PCR within 10 weeks, but significantly more infants had complete testing (i.e. both a birth and a 10-week HIV PCR or a birth HIV PCR only if HIV-positive at birth) in 2020. The mean age at which 10-week HIV PCR was taken remained comparable in both groups: 10.59 weeks in 2019 and 10.68 in 2020 (*P* = 0.549).

**TABLE 2:** HIV PCR uptake.

HIV PCR uptake	Before lockdown (2019)		During lockdown (2020)		Total		*P*
	*n*	%	*n*	%	*n*	%	
**Birth HIV PCR done**	1413	-	1475	-	2888	-	-
No	215	15.2	258	17.5	473	16.4	-
Yes	1198	84.8	1217	82.5	2415	83.6	0.109
**10-week HIV PCR done** †	1407	-	1462	-	2869	-	-
No	558	39.7	432	29.5	990	34.5	-
Yes	849	60.3	1030	70.5	1879	65.5	< 0.001
**Overall HIV PCR done**	1413	-	1475	-	2888	-	-
No recorded testing	132	9.3	137	9.3	269	9.3	0.999
Complete testing‡	705	50.0	849	57.6	1554	53.8	< 0.001
Incomplete testing§	576	40.7	489	33.1	1065	36.9	< 0.001
**HIV PCR done between 2 and 5 weeks**	132	-	137	-	269	-	-
No	105	79.5	110	80.3	215	79.9	-
Yes	27	20.5	27	19.7	54	20.1	0.999

HIV PCR, HIV polymerase chain reaction.

† , Known HIV-positive infants excluded;

‡ , Both a birth and a 10-week HIV PCR or a birth HIV PCR only if HIV-positive at birth;

§ , At least one HIV PCR done: missed either birth or 10-week HIV PCR.

**FIGURE 1:**
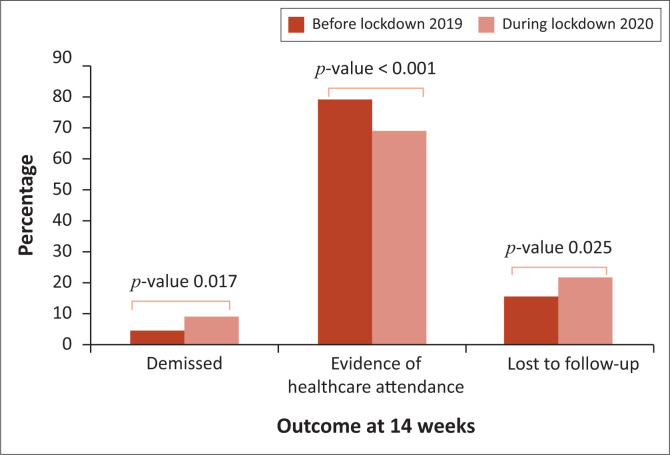
Sub-analysis of infants who did not have 10-week HIV PCR testing.

Sub-analysis of infants who did not have a 10-week HIV PCR (Figure 1) showed that most of these infants had evidence of healthcare attendance in the first 14 weeks of age but were not tested when accessing healthcare. Of those without electronic evidence of interaction with health services, significantly more demised in the 2020 period and significantly more were lost to follow-up.

### Vertical transmission of HIV

Although there was doubling in the proportion of infants with a positive HIV PCR at birth and 10 weeks in the 2020 cohort, the difference did not reach statistical significance (Table 3). There was a nearly threefold (2.8-fold) increase in vertical transmission of HIV by 10 weeks of age in 2020 compared to 2019 (*P* = 0.046).

**TABLE 3:** HIV PCR results.

HIV PCR results	Before lockdown (2019)		During lockdown (2020)		Total		*P*
	*n*	%	*n*	%	*n*	%	
**Birth HIV PCR result**	1198	-	1217		2415	-	-
Negative	1191	99.4	1200	98.6	2391	99.2	-
Positive	6	0.5	13	1.1	19	0.8	0.17
Unknown/inconclusive†	1	0.1	4	0.3	5	0.2	-
**10-week HIV PCR result** ‡	853	-	1042	-	1895	-	-
Negative	845	99.1	1024	98.3	1869	98.6	-
Positive	0	0.0	4	0.4	4	0.2	0.18
Unknown/inconclusive†	8	0.9	14	1.3	22	1.1	-
**Overall HIV PCR**	1308	-	1365	-	2673	-	-
Negative§	1302	99.5	1347	98.7	2649	99.1	-
Positive¶	6	0.5	17	1.2	23	0.9	0.046
Unknown/inconclusive†	0	0.0	1	0.1	1	0.0	-

HIV PCR, HIV polymerase chain reaction.

† , ‘unknown’ denotes HIV PCR tests that did not have a documented result and ‘inconclusive’ denotes an equivocal HIV PCR test result;

‡ , Known HIV-positive infants excluded;

§ , All HIV PCR tests negative;

¶ , Birth or 10 weeks positive HIV PCR test.

### Facility-level data

Facility-level data were only available for birth HIV PCR testing. In both years, facility-level data reflected 100% uptake of birth HIV PCR testing. However, 10-week testing and results were not routinely followed up or recorded by either facility. More HIV PCR tests were recorded in facility-level data than were captured in the electronic database in 2019 and 2020. There were 299 additional HIV birth-PCR tests recorded in facility-based registers in 2020 compared to 7 additional tests recorded in 2019 (Table 4). However, as seen in Table 3, the proportion of infants testing HIV-positive in both years remained comparable to the proportions found in the electronic data set.

**TABLE 4:** Facility-level data.

Birth HIV PCR result	Before lockdown (2019) (*n* = 1205)		During lockdown (2020) (*n* = 1516)		Total (*n* = 2721)		*P*
	*n*	%	*n*	%	*n*	%	
Negative	1196	99.3	1499	98.9	2695	99.0	0.425
Positive	9	0.7	17	1.1	26	1.0	-

HIV PCR, HIV polymerase chain reaction.

## Discussion

This study investigated the effects of the early COVID-19 pandemic and associated lockdown regulations on EID in Cape Town, South Africa. We found a statistically significant increase in vertical transmission of HIV in infants born during the COVID-19 pandemic despite unchanged uptake of birth testing and improved uptake of 10-week testing compared with the same period in the year before. Importantly, of the infants who did not undergo a 10-week HIV PCR more infants demised or were lost to follow-up in the 2020 group.

### HIV PCR uptake

We found that while the HIV PCR uptake at birth was unchanged during the initial COVID-19 lockdown, the 10-week HIV PCR testing uptake improved compared to the same period in the year before, a testament to the resilience of the South African VTP programme.

A recent study looking at the effect of COVID-19 on HIV, tuberculosis and vertical transmission indicators in Mopani district, Limpopo, South Africa, found significant disruptions in HIV testing, ART initiation, and retention in care among children aged 18 months to 14 years. Interestingly, this study did not show any differences in vertical transmission indicators such as the number of antenatal care visits, number of pregnant WLHIV identified and the number of pregnant WLHIV initiated on ART. The reported HIV PCR tests and HIV PCR positive results were also relatively unaffected during the pandemic. There was, however, a marked increase in antenatal care enrolments in May 2020 postulated to be related to women returning to their rural homes after the initial COVID-19 lockdown.^41^ Evidence suggests that this migration of pregnant women back to rural provinces was most pronounced in Limpopo, Mpumalanga and the Northern Cape, with metropolitan areas having a significant decrease in initial antenatal care enrolment visits.^47^

A similar study done in the KwaZulu-Natal province of South Africa, found a small but significant decrease in birth HIV PCR testing during the pandemic but did not investigate differences in vertical transmission.^37^ In both studies, aggregated data from the District Health Information System (DHIS) were used to analyse the absolute number of HIV PCR tests done in a geographical service area. Our data were specific to individual infants who were HIV exposed and followed up for the first 14 weeks of age. Using de-aggregated data allowed us to highlight proportional differences in testing in a defined population rather than differences in the total number of tests done. Differences in the type of data analysed, as well as intrinsic differences in the populations studied such as pre-existing socio-economic differences^48,49^ and impacts of the migration of pregnant women after the initial COVID-19 lockdown may explain differences in the study results.

Despite the gains seen in our study in 10-week HIV PCR testing in 2020, the uptake in both 2019 and 2020 was lower than found in previous studies on EID in the Cape Town Metro by Kalk et al. and Dunning et al., between 2014 and 2015.^9,19^ While being done in policy periods with limited rollout of birth HIV PCR testing, both these studies found the average uptake of HIV PCR testing at 6 to 10 weeks at over 80%. Given the higher proportion of birth HIV PCR testing in our study, this relatively lower uptake of follow-up testing is consistent with their findings reporting that infants who undergo birth HIV PCR testing are less likely to present for follow-up testing.^9,19^ Kalk et al. also found that infants classified as at high risk for HIV transmission and those born to mothers with inadequate antenatal ART were also less likely to present for follow-up testing – suggesting that pre-existing patterns of poor health-seeking behaviour may contribute to decreased uptake of EID.^9^

These results differ from a more recently published study which found that having a birth HIV PCR was associated with an increased uptake of repeat HIV PCR testing at 6 or 10 weeks in a cohort of infants born in Khayelitsha, Cape Town.^50^ While this study spanned a similar time period as the Kalk et al. and Dunning et al. studies, there were several differences in study methodology. Whereas our study, as well as those by Kalk et al. and Dunning et al., analysed routinely collected data and testing results, the Khayelitsha study employed study nurses to take blood samples for both birth HIV PCR samples and point-of-care HIV testing, communicate results, and counsel mothers on the further need for infant testing, prophylaxis and follow-up. A full-time patient tracer also monitored the National Health Laboratory Service (NHLS) database for evidence of 6/10-week HIV PCR testing and if no testing was evident one month after testing was due, patients were actively traced and reminded about follow-up testing.^50^ These differences in study methodology including additional testing, active counselling and tracing of study participants may explain differences found in uptake of repeat HIV PCR testing.

Another large study in Johannesburg, Gauteng, South Africa, analysed data extracted from the NHLS database from October 2018 to September 2021 and determined trends in the total number of tests performed and the total number of children with HIV diagnosed, stratified by age, and compared these data to EID indicators collected by the DHIS. During the COVID-19 pandemic they found much higher rates of birth and 10-week HIV PCR uptake than in our study, with the number of HIV PCR tests done at birth and 10 weeks closely approximating the number of live births to WLHIV. While a 6% decrease in 10-week HIV PCR uptake was noted during the second and third quarter of 2020, the period with the strictest COVID-19 lockdown, overall, there was an increase in HIV testing across all age groups over the three 12-month periods analysed.^40^

Although also using data from a centralised database and analysing routinely collected data, the Johannesburg study had a much broader definition of a birth HIV PCR test (any HIV PCR done from birth up to 6 weeks) and the total number of birth tests performed was found to be higher than the total live births to WLHIV, postulated to be due to a combination of under-reporting of the DHIS indicators and repeat HIV PCR tests performed in the first 6 weeks of life. The absence of unique patient identifiers in the cohort studied may have further impacted on duplication of testing.^40^

The improved 10-week HIV PCR testing uptake in 2020 found in our study, and the even higher uptake found in Khayelitsha and Johannesburg cohorts, clearly highlight the resilience of the South African VTP programme – even in the face of a global pandemic. Understanding the drivers behind the higher uptake of testing in 2020 could inform interventions to further improve uptake of the follow-up testing and could help to strengthen initiatives targeting uptake of other routine child health services such as childhood immunisation, which suffered severe disruptions during the COVID-19 lockdown.

The increase we found in infant mortality and loss to follow-up in the 2020 group of infants that did not have a 10-week HIV PCR suggests that the COVID-19 pandemic and associated lockdown restrictions did have a significant impact on children’s access to healthcare. However, in both years, most infants who did not have a 10-week HIV PCR did have evidence of healthcare attendance. This finding is equally important and represents critical missed opportunities – perhaps an argument for integrated packages of care with VTP interventions forming part of other routine child health visits.

### Vertical transmission

Although there were no statistically significant changes in birth HIV PCR positivity rates between the two groups in our study, overall HIV transmission by 10 weeks of age more than doubled, a notable change from the steady decline in vertical transmission over the last decade.^14,15,16,17^ In contrast to our findings, the Johannesburg study found there was an overall decrease in positive HIV PCR tests in children < 2 years old in their 3-year study period. Interestingly, they did note some variations during the initial stages of lockdown restrictions; despite no obvious differences in the number of HIV PCR tests performed during the initial strictest COVID-19 lockdown, they found a sudden decrease in the number of positive birth and 10-week HIV PCR tests in that period, followed by a subsequent increase in the number of positive tests when restrictions were relaxed. While the cohort studied was very large and their data sources robust, there were some reported data discrepancies between the two major data sources used in this study that may have impacted on their results. These discrepancies were most notable in relation to the number of positive HIV PCRs found at birth and 10 weeks and were possibly related to the inability to link patient tests accurately as unique patient identifying numbers were not used in that population.^40^

Our findings of increased vertical transmission of HIV in the first wave of the pandemic coupled with data showing a decrease in antenatal care enrolment^29,51,52^ and ART collection^30,36^ suggests service disruption with decreased access and adherence to maternal ART during the COVID-19 lockdown potentially impacting vertical transmission rates. This is a powerful reminder that infant health is inextricably linked with maternal health and that interventions targeting and championing child health also need to consider maternal health. Increased HIV transmission has far-reaching impacts and could undermine hard-won gains in child health and child mortality made over many years. There are also implications for forward planning and the need to increase capacity for healthcare services for children living with HIV.

### Differences between electronic data and facility records

In the electronic data analysed, we found that more than 80% of infants in both cohorts underwent birth HIV PCR testing. This is higher than previous estimates of birth testing uptake,^9,19^ but still falls well below the national 95/95/95 targets. Notably, facility-level data from paper registers did not correspond with the electronic data. Although rates of vertical transmission were comparable, absolute number of birth HIV PCR tests recorded as aggregate data by the birth facilities were higher than the number of birth HIV PCR tests reflected in the electronic data set, and uptake of birth HIV PCR testing was much higher in facility-based registers. The discrepancy was much more pronounced in the 2020 group, which may indicate that electronic data collection was impacted by the COVID-19 pandemic and lockdown disruptions. Exploring these data discrepancies may identify underlying problems in testing or data capture procedures that contribute to reported birth testing rates remaining persistently below national targets. Further research into the impacts of staff re-allocation or absence during the pandemic is warranted to determine if this may have affected data collection accuracy.

## Strengths and limitations

Our study was retrospective and thus limited by the accuracy of data collected and entered into existing registers and electronic databases. A recent study evaluating the quality of data from the NHLS data warehouse found poor-quality demographic data, particularly for infants and young children,^53^ highlighting potential limitations in using electronic databases which may have impacted the study.

The focus of our study was limited to EID and did not explore other aspects of the VTP cascade or linkage to care for HIV-positive infants. The study also did not include data on mode of delivery, predelivery maternal viral load, ART duration and/or interruption which may all impact transmission rates. The single geographic service area data may not be generalisable due to differences in healthcare service infrastructure and accessibility. Although the overall number of deliveries at study facilities remained constant, we did not investigate potential differences in delivery patterns, such as rates of preterm deliveries, caesarean sections, etc. that may have been influenced by the lockdown restrictions. Sample bias may have occurred as all infants in our study were born in regional level referral hospitals within a metropolitan city.

## Conclusion and recommendations

The uptake of routine HIV PCR testing of HIV-exposed infants increased during the COVID-19 lockdown in 2020, highlighting the resilience of the South African VTP programme – even in the face of a global pandemic. However, higher rates of vertical transmission and the high proportion of loss to follow-up or demise among infants not tested at 10 weeks in 2020, supports the hypothesis that the COVID-19 pandemic lockdown did have an impact on health services and access to care.

Further research to unpack the drivers behind the increased HIV PCR uptake in 2020 could inform interventions for future pandemics and bolster strategies to improve retention of mother-infant dyads in the VTP cascade. More research is also needed to determine if the increases in vertical transmission seen in this study have continued following the lifting of the COVID-19 lockdown restrictions. This would help in interrogating child health service vulnerabilities in times of national crisis.
